# An unusual case of penetrating ocular trauma with a pressure cooker

**DOI:** 10.4103/0974-620X.64234

**Published:** 2010

**Authors:** Soumya Swarup Chattopadhyay, Udayaditya Mukhopadhyay, Kumar Saurabh

**Affiliations:** Regional Institute of Ophthalmology, Medical College, Kolkata, West Bengal, India

**Keywords:** Domestic, ocular trauma, pressure cooker, women

## Abstract

Ocular trauma is a major cause of vision loss. The circumstances and agents implicated in such injuries are diverse. We present an unusual case of penetrating ocular trauma with the nozzle of a pressure cooker lid in a 32-year-old housewife causing deep laceration of the upper eye lid and sclera. The impacted metallic nozzle was removed on an emergency basis. Autoevisceration of ocular contents due the high velocity impact resulted in the final decision to surgically complete the evisceration and implant a glass ball implant. This case highlights the propensity of grievous ocular trauma in a domestic environment.

## Introduction

Ocular trauma is a leading cause of vision loss.[1,2] Injury sustained at workplace among men is the commonest scenario of such blinding ocular trauma.[3,4] Domestic ocular injuries among women and children are of no lesser magnitude but such cases are probably underreported.[2,5,6] We present an unusual case of penetrating ocular trauma in a housewife with the nozzle of the pressure cooker lid while working in kitchen.

## Case Report

A 32-year-old housewife presented to our emergency with injury to her right eye due to pressure cooker burst. She was using a kerosene stove to cook and was sitting beside it keeping the pressure cooker below the level of her head. When the excessive pressure build up pushed off the safety valve of the pressure cooker, she tried to replace it. During that act, the nozzle of the pressure cooker lid also gave away and hit the right side of her face like a missile.

**Figure 1:**
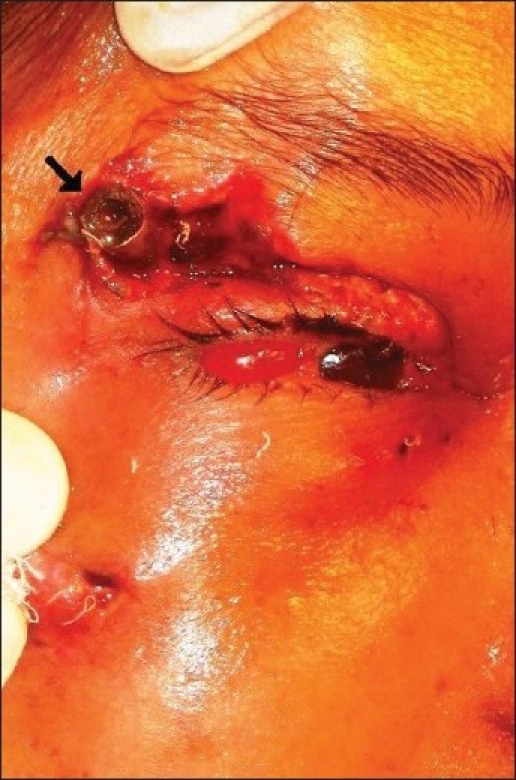
Impacted pressure cooker nozzle (arrow) in the right eye

**Figure 2:**
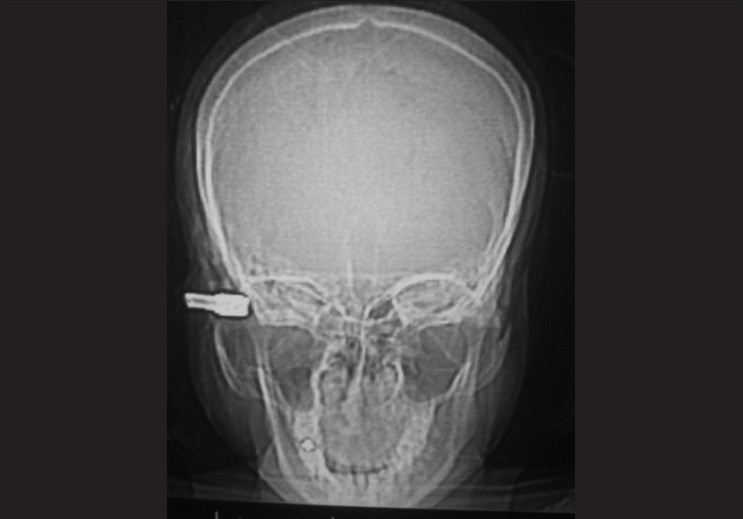
Computed tomography scan image showing impacted nozzle, with intact bony architecture

**Figure 3:**
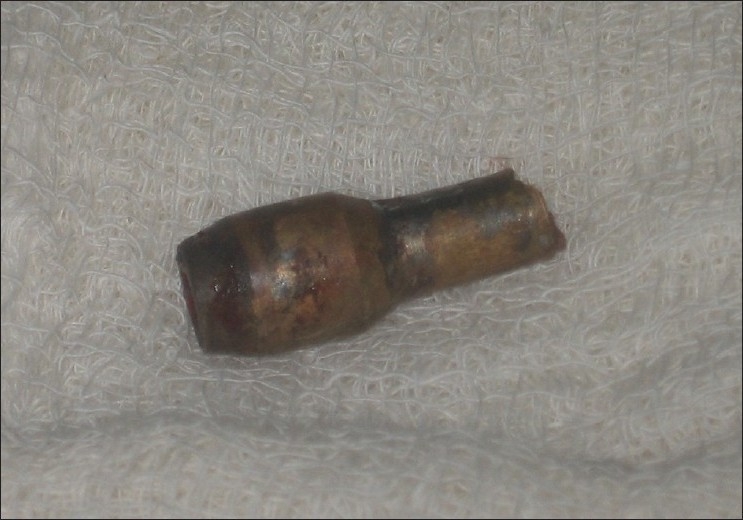
Metallic nozzle of the lid of the pressure cooker lid

Visual acuity in the emergency room was reduced to light perception. With a pulse rate of 90/minute, blood pressure 140/80 mm Hg and Glassgow coma scale of 13, she was hemodynamically stable. She was administered tetanus toxoid by her family physician prior to coming to us. A deep laceration was noted between the right upper eye lid crease and temporal end of the eye brow. The metallic nozzle was seen impacted in the laceration site [Figure 1]. There was a small laceration on the cheek on the same side. The eye showed extensive chemosis, total hyphema and hypotony. Computed tomography image of brain and orbit showed disorganized globe with no evidence of bony or intracranial injury [Figure 2]. The visual prognosis and possibility of intraoperative encounter to an irreversibly damaged eye, needing evisceration was explained to the nearest relatives of the patient. The impacted nozzle was removed in the emergency operation theatre under general anaesthesia [Figure 3]. Examination of the eye on the operating table revealed a full-thickness scleral laceration above the insertion of lateral rectus. The globe was hypotonous due to autoevisceration of the ocular contents. Evisceration was completed and scleral laceration was repaired with a 6-0 vicryl suture. Cost restrictions compelled us to use a glass ball implant to replace the orbital volume in our patient instead of hydroxyapatite implant. The lid laceration was repaired with 6-0 vicryl suture. Patient was maintained on intravenous ceftriaxone (1 g every 12 h) and intravenous gentamicin (80 mg every 12 h) systemically along with topical fortified cefazolin (50 mg/ml).

## Discussion

Penetrating ocular trauma is a leading cause of blindness.[1,2] Though injury at workplace are more common, those at domestic environment are equally severe.[5] Since women and children are more likely to sustain blinding ocular injury at home, there is a need to implement eye health awareness programs targeted at increasing the awareness about safety measures at home.[5,7,8] In our patient, not leaning over the pressure cooker, releasing the pressure, and replacing the safety valve, would have saved the eye sight of the patient. Similarly regular cleaning of the pressure cooker valve and changing of the rubber seal at frequent intervals would go long way in averting such an accident. Most of the domestic ocular injuries are mechanical in nature.[9] Therefore correct positioning household equipments and right posture during work can also prove effective in reducing such injuries.

Domestic ocular injuries are usually caused by fist, stick, balls, fire crackers, bursting bottles, needles and other sharp objects.[5] A previous study of causes of ocular trauma had stated pressure cooker explosion as the cause in 4.6% of cases.[10] In our case, the pressure cooker nozzle virtually acted as bullet forcing us to consider possible traumatic brain injury. A conscious and cooperative patient and lack of bony injury at the site of impact, as seen in computed tomography scan assured us otherwise. Examination of the globe and primary removal of the foreign body was undertaken under general anaesthesia to minimize possible iatrogenic trauma to the ocular tissue. However autoevisceration of the ocular contents left us with no choice but to complete the evisceration and implant a glass ball.
